# Supplementary material for: Teaching hospitals and their influence on survival after valve replacement procedures: A retrospective cohort study using inverse probability of treatment weighting (IPTW)
Source: PLoS One. 2023 Aug 25;18(8):e0290734. doi: 10.1371/journal.pone.0290734 (PMC10456128; doi:10.1371/journal.pone.0290734)
Supplement: S1 Table — (PDF) [file pone.0290734.s001.pdf]

S1 Table. Region definitions.

Region	Departments
**Atlantic**	Atlántico, Bolivar, Cesar, Cordoba, Magdalena
**Bogota**	-
**Central**	Antioquia, Caldas, Caquetá, Huila, Quindío, Risaralda, Tolima
**Eastern**	Boyacá, Cundinamarca, Meta, Norte de Santander, Santander
**Pacific**	Chocó, Valle del Cauca, Cauca, Nariño
**Orinoco-Amazonia**	Arauca, Casanare, Meta, Vichada, Guaviare, Amazonas, Caquetá, Guainía, Guaviare, Putumayo, Vaupés
