# Supplementary material for: Teaching hospitals and their influence on survival after valve replacement procedures: A retrospective cohort study using inverse probability of treatment weighting (IPTW)
Source: PLoS One. 2023 Aug 25;18(8):e0290734. doi: 10.1371/journal.pone.0290734 (PMC10456128; doi:10.1371/journal.pone.0290734)
Supplement: S2 Table — (PDF) [file pone.0290734.s002.pdf]

S2 Table. Frequencies of valve replacement procedures and other concomitant cardiovascular surgical procedures performed simultaneously with the "index procedure" and frequencies of valve reinterventions.

Index procedure [a]							
**VR Procedures**	**TH (n=1,073)**	**Non-TH (n=2,526)**	**Overall (N=3,599)**	**Other Procedures**	**TH (n=219)**	**Non-TH (n=604)**	**Overall (N=823)**
Aortic SVR	597 (55.6%)	1515 (60.0%)	2112 (58.7%)	CABG	107 (48.9%)	234 (38.7%)	341 (41.4%)
Mitral SVR	283 (26.4%)	656 (26.0%)	939 (26.1%)	Aorta surgery	52 (23.7%)	239 (39.6%)	291 (35.4%)
Aortic TVR	74 (6.9%)	189 (7.5%)	263 (7.3%)	HTA/R	34 (15.5%)	60 (9.9%)	94 (11.4%)
Aortic MIVR	69 (6.4%)	37 (1.5%)	106 (2.9%)	Tricuspid SVr	13 (5.9%)	35 (5.8%)	48 (5.8%)
Tricuspid SVR	14 (1.3%)	57 (2.3%)	71 (2.0%)	Mitral SVr	6 (2.7%)	23 (3.8%)	29 (3.5%)
Mitral TVR	7 (0.7%)	37 (1.5%)	44 (1.2%)	ASD/VSD Surgery	4 (1.8%)	8 (1.3%)	12 (1.5%)
Mitral MIVR	17 (1.6%)	9 (0.4%)	26 (0.7%)	Aortic SVr	2 (0.9%)	1 (0.2%)	3 (0.4%)
Ross Procedure	3 (0.3%)	21 (0.8%)	24 (0.7%)	Aortic TVr	0 (0.0%)	3 (0.5%)	3 (0.4%)
Pulmonary SVR	7 (0.7%)	1 (0.0%)	8 (0.2%)	Pulmonary SVr	0 (0.0%)	1 (0.2%)	1 (0.1%)
Other*	2 (0.2%)	4 (0.2%)	6 (0.2%)	Tricuspid MIVr	1 (0.5%)	0 (0.0%)	1 (0.1%)
**Valve reintervention [a]**							
**VR Procedures**	**TH (n=46)**	**Non-TH (n=78)**	**Overall (N=124)**	**Vr Procedures**	**TH (n=0)**	**Non-TH (n=3)**	**Overall (N=3)**
Aortic SVR	32 (69.6%)	41 (52.6%)	73 (58.9%)	Aortic TVr	0 (0.0%)	1 (33.3%)	1 (33.3%)
Mitral SVR	7 (15.2%)	26 (32.1%)	34 (27.4%)	Mitral SVr	0 (0.0%)	1 (33.3%)	1 (33.3%)
Tricuspid SVR	0 (0.0%)	6 (7.7%)	6 (4.8%)	Tricuspid SVr	0 (0.0%)	1 (33.3%)	1 (33.3%)
Aortic TVR	1 (2.2%)	3 (3.8%)	4 (3.2%)	-	-	-	-
Aortic MIVR	3 (6.5%)	0 (0.0%)	3 (2.4%)	-	-	-	-
Mitral MIVR	2 (4.4%)	1 (1.3%)	2 (1.6%)	-	-	-	-
Ross Procedure	0 (0.0%)	1 (1.3%)	1 (0.8%)	-	-	-	-
Mitral TVR	1 (2.2%)	0 (0.0%)	1 (0.8%)	-	-	-	-

[a] One single patient may have more than one kind of valve index procedure or reintervention. ASD/VSD: Atrial/Ventricular Septum Defect; CABG: Coronary Artery Bypass Graft, HTA/R: Heart tissue ablation / resection; MIVR: Minimally Invasive Valve Replacement; MIVr: Minimally Invasive Valve repair; SVR: Surgical Valve Replacement; SVr: Surgical Valve repair; TH: Teaching Hospital; TVR: Transcatheter Valve Replacement; TVr: Transcatheter Valve repair; Vr: Valve repair; VR: Valve Replacement. *Other: Pulmonary TVR, Tricuspid MIVR, Tricuspid TVR.
