# Supplementary material for: Teaching hospitals and their influence on survival after valve replacement procedures: A retrospective cohort study using inverse probability of treatment weighting (IPTW)
Source: PLoS One. 2023 Aug 25;18(8):e0290734. doi: 10.1371/journal.pone.0290734 (PMC10456128; doi:10.1371/journal.pone.0290734)
Supplement: S3 Table — (PDF) [file pone.0290734.s003.pdf]

S3 Table. Cumulative incidences of death at 30 days, 90 days, and one year per teaching hospital status.

	30-day status				90-day status				One-year status			
	TH		Non-TH		TH		Non-TH		TH		Non-TH	
	Dead (n=29)	Alive (n=1,022)	Dead (n=180)	Alive (n=2,286)	Dead (n=46)	Alive (n=1,005)	Dead (n=234)	Alive (n=2,232)	Dead (n=82)	Alive (n=969)	Dead (n=301)	Alive (n=2,165)
**Age Group**												
≤ 60	4 (1.1%)	372 (98.9%)	49 (5.0%)	929 (95.0%)	10 (2.7%)	366 (97.3%)	57 (5.8%)	921 (94.2%)	17 (4.5%)	359 (95.5%)	70 (7.2%)	908 (92.8%)
60-70	7 (2.3%)	299 (97.7%)	61 (8.7%)	643 (91.3%)	10 (3.3%)	296 (96.7%)	79 (11.2%)	625 (88.8%)	20 (6.5%)	286 (93.5%)	99 (14.1%)	605 (85.9%)
70-80	12 (4.0%)	287 (96.0%)	58 (9.5%)	554 (90.5%)	18 (6.0%)	281 (94.0%)	80 (13.1%)	532 (86.9%)	30 (10.0%)	269 (90.0%)	102 (16.7%)	510 (83.3%)
≥ 80	6 (8.6%)	64 (91.4%)	12 (7.0%)	160 (93.0%)	8 (11.4%)	62 (88.6%)	18 (10.5%)	154 (89.5%)	15 (21.4%)	55 (78.6%)	30 (17.4%)	142 (82.6%)
**Sex**												
Male	16 (2.6%)	589 (97.4%)	104 (6.9%)	1,411 (93.1%)	24 (4.0%)	581 (96.0%)	138 (9.1%)	1,377 (90.9%)	47 (7.8%)	558 (92.2%)	181 (11.9%)	1,334 (88.1%)
Female	13 (2.9%)	433 (97.1%)	76 (8.0%)	875 (92.0%)	22 (4.9%)	424 (95.1%)	96 (10.1%)	855 (89.9%)	35 (7.8%)	411 (92.2%)	120 (12.6%)	831 (87.4%)
**CCI - Categories**												
None (0)	1 (0.4%)	279 (99.6%)	27 (4.4%)	590 (95.6%)	3 (1.1%)	277 (98.9%)	36 (5.8%)	581 (94.2%)	10 (3.6%)	270 (96.4%)	45 (7.3%)	572 (92.7%)
Mild (1-2)	16 (3.6%)	433 (96.4%)	68 (6.5%)	974 (93.5%)	22 (4.9%)	427 (95.1%)	89 (8.5%)	953 (91.5%)	35 (7.8%)	414 (92.2%)	113 (10.8%)	929 (89.2%)
Moderate (3-4)	5 (2.4%)	200 (97.6%)	41 (8.6%)	433 (91.4%)	10 (4.9%)	195 (95.1%)	55 (11.6%)	419 (88.4%)	18 (8.8%)	187 (91.2%)	74 (15.6%)	400 (84.4%)
Severe (≥5)	7 (6.0%)	110 (94.0%)	44 (13.2%)	289 (86.8%)	11 (9.4%)	106 (90.6%)	54 (16.2%)	279 (83.8%)	19 (16.2%)	98 (83.8%)	69 (20.7%)	264 (79.3%)
**Region**												
Bogota	24 (3.3%)	694 (96.7%)	47 (7.4%)	588 (92.6%)	40 (5.6%)	678 (94.4%)	58 (9.1%)	577 (90.9%)	67 (9.3%)	651 (90.7%)	73 (11.5%)	562 (88.5%)
Central	4 (1.3%)	293 (98.7%)	57 (7.6%)	697 (92.4%)	5 (1.7%)	292 (98.3%)	75 (9.9%)	679 (90.1%)	13 (4.4%)	284 (95.6%)	91 (12.1%)	663 (87.9%)
Other*	1 (2.8%)	35 (97.2%)	76 (7.1%)	1,001 (92.9%)	1 (2.8%)	35 (97.2%)	101 (9.4%)	976 (90.6%)	2 (5.6%)	34 (94.4%)	137 (12.7%)	940 (87.3%)
**Weight of procedure**												
Isolated valve procedure	18 (2.2%)	816 (97.8%)	113 (6.1%)	1,752 (93.9%)	26 (3.1%)	808 (96.9%)	149 (8.0%)	1,716 (92.0%)	56 (6.7%)	778 (93.3%)	194 (10.4%)	1,671 (89.6%)
Double valve procedure	0 (0.0%)	19 (100.0%)	6 (13.3%)	39 (86.7%)	0 (0.0%)	19 (100.0%)	7 (15.6%)	38 (84.4%)	1 (5.3%)	18 (94.7%)	8 (17.8%)	37 (82.2%)
Isolated valve + 1 procedure	8 (4.5%)	169 (95.5%)	56 (11.1%)	447 (88.9%)	17 (9.6%)	160 (90.4%)	70 (13.9%)	433 (86.1%)	22 (12.4%)	155 (87.6%)	91 (18.1%)	412 (81.9%)
Double valve + ≥2 procedures	1 (33.3%)	2 (66.7%)	1 (7.1%)	13 (92.9%)	1 (33.3%)	2 (66.7%)	2 (14.3%)	12 (85.7%)	1 (33.3%)	2 (66.7%)	2 (14.3%)	12 (85.7%)
Isolated valve + ≥2 procedures	2 (11.1%)	16 (88.9%)	4 (10.3%)	35 (89.7%)	2 (11.1%)	16 (88.9%)	6 (15.4%)	33 (84.6%)	2 (11.1%)	16 (88.9%)	6 (15.4%)	33 (84.6%)
**Technique**												
Surgical	27 (3.0%)	861 (97.0%)	160 (7.3%)	2,034 (92.7%)	42 (4.7%)	846 (95.3%)	210 (9.6%)	1,984 (90.4%)	69 (7.8%)	819 (92.2%)	260 (11.9%)	1,934 (88.1%)
Transcatheter	1 (1.2%)	80 (98.8%)	17 (7.5%)	210 (92.5%)	3 (3.7%)	78 (96.3%)	21 (9.3%)	206 (90.7%)	10 (12.3%)	71 (87.7%)	37 (16.3%)	190 (83.7%)
Minimally Invasive	1 (1.2%)	81 (98.8%)	3 (6.7%)	42 (93.3%)	1 (1.2%)	81 (98.8%)	3 (6.7%)	42 (93.3%)	3 (3.7%)	79 (96.3%)	4 (8.9%)	41 (91.1%)

CCI: Charlson Comorbidity Index; TH: Teaching Hospital. *Other (region): Atlantic, Eastern, and Pacific.
