# Supplementary material for: Teaching hospitals and their influence on survival after valve replacement procedures: A retrospective cohort study using inverse probability of treatment weighting (IPTW)
Source: PLoS One. 2023 Aug 25;18(8):e0290734. doi: 10.1371/journal.pone.0290734 (PMC10456128; doi:10.1371/journal.pone.0290734)
Supplement: S4 Table — (PDF) [file pone.0290734.s004.pdf]

S4 Table. Cumulative incidences of death at 30 days, 90 days, and one year per index procedure.

	30-days Status		90-days Status		One year Status	
	Dead	Alive	Dead	Alive	Dead	Alive
**Aortic**						
Aortic SVR	113 (5.4%)	1,999 (94.6%)	151 (7.1%)	1,961 (92.9%)	201 (9.5%)	1,911 (90.5%)
Aortic MIVR	4 (3.9%)	99 (96.1%)	4 (3.9%)	99 (96.1%)	7 (6.8%)	96 (93.2%)
Aortic TVR	17 (6.5%)	245 (93.5%)	23 (8.8%)	239 (91.2%)	41 (15.6%)	221 (84.4%)
**Mitral**						
Mitral SVR	75 (8.0%)	864 (92.0%)	103 (11.0%)	836 (89.0%)	128 (13.6%)	811 (86.4%)
Mitral MIVR	0 (0.0%)	26 (100.0%)	0 (0.0%)	26 (100.0%)	0 (0.0%)	26 (100.0%)
Mitral TVR	1 (2.3%)	43 (97.7%)	1 (2.3%)	43 (97.7%)	6 (13.6%)	38 (86.4%)
**Tricuspid**						
Tricuspid SVR	5 (7.0%)	66 (93.0%)	6 (8.5%)	65 (91.5%)	8 (11.3%)	63 (88.7%)
Tricuspid MIVR	0 (0.0%)	1 (100.0%)	0 (0.0%)	1 (100.0%)	0 (0.0%)	1 (100.0%)
Tricuspid TVR	0 (0.0%)	1 (100.0%)	0 (0.0%)	1 (100.0%)	0 (0.0%)	1 (100.0%)
**Pulmonary**						
Pulmonary SVR	0 (0.0%)	8 (100.0%)	0 (0.0%)	8 (100.0%)	0 (0.0%)	8 (100.0%)
Pulmonary TVR	0 (0.0%)	4 (100.0%)	0 (0.0%)	4 (100.0%)	0 (0.0%)	4 (100.0%)
**Ross**						
Ross procedure	2 (8.3%)	22 (91.7%)	2 (8.3%)	22 (91.7%)	3 (12.5%)	21 (87.5%)

MIVR: Minimally Invasive Valve Replacement; SVR: Surgical Valve Replacement; TVR: Transcatheter Valve Replacement; VR: Valve Replacement. Since one single patient may have more than one kind of valve index procedure, in the event of e.g., a patient with a MIVR or a TVR converted to SVR, the SVR is the index procedure.
