# Supplementary material for: Teaching hospitals and their influence on survival after valve replacement procedures: A retrospective cohort study using inverse probability of treatment weighting (IPTW)
Source: PLoS One. 2023 Aug 25;18(8):e0290734. doi: 10.1371/journal.pone.0290734 (PMC10456128; doi:10.1371/journal.pone.0290734)
Supplement: S5 Table — (PDF) [file pone.0290734.s005.pdf]

S5 Table. Cumulative incidences of 90-day readmission and one-year reintervention per teaching hospital status.

	90-days Status				One-year Status			
	TH		Non-TH		TH		Non-TH	
	No readmission (N=812)	Readmission (N=239)	No readmission (N=1,877)	Readmission (N=589)	No reintervention (N=1,005)	Reintervention (N=46)	No reintervention (N=2,385)	Reintervention (N=81)
**Age Group**								
≤ 60	306 (81.4%)	70 (18.6%)	753 (77.0%)	225 (23.0%)	355 (94.4%)	21 (5.6%)	944 (96.5%)	34 (3.5%)
60-70	240 (78.4%)	66 (21.6%)	528 (75.0%)	176 (25.0%)	292 (95.4%)	14 (4.6%)	679 (96.4%)	25 (3.6%)
70-80	223 (74.6%)	76 (25.4%)	463 (75.7%)	149 (24.3%)	289 (96.7%)	10 (3.3%)	598 (97.7%)	14 (2.3%)
≥ 80	43 (61.4%)	27 (38.6%)	133 (77.3%)	39 (22.7%)	69 (98.6%)	1 (1.4%)	164 (95.3%)	8 (4.7%)
**Sex**								
Male	465 (76.9%)	140 (23.1%)	1,162 (76.7%)	353 (23.3%)	581 (96.0%)	24 (4.0%)	1,467 (96.8%)	48 (3.2%)
Female	347 (77.8%)	99 (22.2%)	715 (75.2%)	236 (24.8%)	424 (95.1%)	22 (4.9%)	918 (96.5%)	33 (3.5%)
**CCI - Categories**								
None (0)	225 (80.4%)	55 (19.6%)	493 (79.9%)	124 (20.1%)	265 (94.6%)	15 (5.4%)	590 (95.6%)	27 (4.4%)
Mild (1-2)	351 (78.2%)	98 (21.8%)	787 (75.5%)	255 (24.5%)	432 (96.2%)	17 (3.8%)	1,010 (96.9%)	32 (3.1%)
Moderate (3-4)	158 (77.1%)	47 (22.9%)	344 (72.6%)	130 (27.4%)	195 (95.1%)	10 (4.9%)	465 (98.1%)	9 (1.9%)
Severe (≥5)	78 (66.7%)	39 (33.3%)	253 (76.0%)	80 (24.0%)	113 (96.6%)	4 (3.4%)	320 (96.1%)	13 (3.9%)
**Region**								
Bogota	547 (76.2%)	171 (23.8%)	458 (72.1%)	177 (27.9%)	681 (94.8%)	37 (5.2%)	612 (96.4%)	23 (3.6%)
Central	242 (81.5%)	55 (18.5%)	598 (79.3%)	156 (20.7%)	290 (97.6%)	7 (2.4%)	729 (96.7%)	25 (3.3%)
Other*	23 (63.9%)	13 (36.1%)	821 (76.2%)	256 (23.8%)	34 (94.4%)	2 (5.6%)	1,044 (96.9%)	33 (3.1%)
**Weight of procedure**								
Isolated valve procedure	660 (79.1%)	174 (20.9%)	1,469 (78.8%)	396 (21.2%)	793 (95.1%)	41 (4.9%)	1,799 (96.5%)	66 (3.5%)
Double valve procedure	15 (78.9%)	4 (21.1%)	30 (66.7%)	15 (33.3%)	18 (94.7%)	1 (5.3%)	44 (97.8%)	1 (2.2%)
Isolated valve + 1 procedure	124 (70.1%)	53 (29.9%)	351 (69.8%)	152 (30.2%)	173 (97.7%)	4 (2.3%)	491 (97.6%)	12 (2.4%)
Double valve + ≥2 procedures	3 (100.0%)	0 (0.0%)	6 (42.9%)	8 (57.1%)	3 (100.0%)	0 (0.0%)	13 (92.9%)	1 (7.1%)
Isolated valve + ≥2 procedures	10 (55.6%)	8 (44.4%)	21 (53.8%)	18 (46.2%)	18 (100.0%)	0 (0.0%)	38 (97.4%)	1 (2.6%)
**Technique**								
Surgical	690 (77.7%)	198 (22.3%)	1,660 (75.7%)	534 (24.3%)	846 (95.3%)	42 (4.7%)	2,117 (96.5%)	77 (3.5%)
Transcatheter	59 (72.8%)	22 (27.2%)	178 (78.4%)	49 (21.6%)	80 (98.8%)	1 (1.2%)	223 (98.2%)	4 (1.8%)
Minimally Invasive	63 (76.8%)	19 (23.2%)	39 (86.7%)	6 (13.3%)	79 (96.3%)	3 (3.7%)	45 (100.0%)	0 (0.0%)

CCI: Charlson Comorbidity Index; TH: Teaching Hospital. *Other (region): Atlantic, Eastern, and Pacific.
