# Supplementary material for: Teaching hospitals and their influence on survival after valve replacement procedures: A retrospective cohort study using inverse probability of treatment weighting (IPTW)
Source: PLoS One. 2023 Aug 25;18(8):e0290734. doi: 10.1371/journal.pone.0290734 (PMC10456128; doi:10.1371/journal.pone.0290734)
Supplement: S6 Table — (PDF) [file pone.0290734.s006.pdf]

S6 Table. **Baseline characteristics before and after inverse probability of treatment weighting with restriction.**

	Unweighted sample			Weighted sample		
	Non-TH (n=2,466)	TH (n=1,051)	SMD	Non-TH (n=1,935.6)	TH (n=1,889.5)	SMD
**Age Group**			0.096			0. 070
≤ 60	978 (39.7)	376 (35.8)		777.6 (40.2)	709.6 (37.6)	
60-70	704 (28.5)	306 (29.1)		568.2 (29.4)	554.9 (29.4)	
70-80	612 (24.8)	299 (28.4)		454.5 (23.5)	496.3 (26.3)	
≥ 80	172 (7.0)	70 (6.7)		135.2 (7.0)	128.8 (6.8)	
**Sex**						
Female	951 (38.6)	446 (42.4)	0.079	807.7 (41.7)	773.6 (40.9)	0. 016
**CCI – Categories**			0.075			0. 025
None (0)	617 (25.0)	280 (26.6)		482.1 (24.9)	452.2 (23.9)	
Mild (1-2)	1,042 (42.3)	449 (42.7)		819.8 (42.4)	801.3 (42.4)	
Moderate (3-4)	474 (19.2)	205 (19.5)		401.6 (20.7)	404.0 (21.4)	
Severe (≥5)	333 (13.5)	117 (11.1)		232.1 (12.0)	232.0 (12.3)	
**Weight of procedure**			0.095			0. 027
Isolated Valve Procedure	1,865 (75.6)	834 (79.4)		1,550.1 (80.1)	1,524.5 (80.7)	
Other Combined*	587 (23.8)	214 (20.4)		373.0 (19.3)	349.7 (18.5)	
Double Valve + ≥1 Procedures	14 (0.6)	3 (0.3)		12.5 (0.6)	15.3 (0.8)	
**Technique**			0.284			0. 009
Surgical	2,194 (89.0)	888 (84.5)		1,671.8 (86.4)	1,636.6 (86.6)	
Transcatheter	227 (9.2)	81 (7.7)		167.3 (8.6)	162.2 (8.6)	
Minimally Invasive	45 (1.8)	82 (7.8)		96.4 (5.0)	90.8 (4.8)	
**Year of surgery**			0.201			0. 071
2016-2017	818 (33.2)	300 (28.5)		540.7 (27.9)	518.2 (27.4)	
2018	880 (35.7)	478 (45.5)		810.9 (41.9)	852.3 (45.1)	
2019	768 (31.1)	273 (26.0)		583.9 (30.2)	519.1 (27.5)	
**Region**			1.212			0. 025
Bogota	635 (25.8)	718 (68.3)		1,191.6 (61.6)	1,151.0 (60.9)	
Central	754 (30.6)	297 (28.3)		655.2 (33.9)	642.4 (34.0)	
Other**	1077 (43.7)	36 (3.4)		88.7 (4.6)	96.1 (5.1)	
**Insurer**			1.241			0.076
Insurer 1	45 (1.8)	315 (30.0)		308.8 (16.0)	303.6 (16.1)	
Insurer 2	821 (33.3)	293 (27.9)		365.5 (18.9)	380.2 (20.1)	
Insurer 3	350 (14.2)	21 (2.0)		89.8 (4.6)	61.4 (3.2)	
Insurer 4	308 (12.5)	303 (28.8)		581.7 (30.1)	563.2 (29.8)	
Other***	942 (38.2)	119 (11.3)		589.7 (30.5)	581.3 (30.8)	

CCI: Charlson Comorbidity Index; SMD: Standardized Mean Difference; TH: Teaching Hospital; Other Combined*: Double Valve Procedure, Isolated Valve + 1 Procedure, Isolated Valve + ≥2 Procedures; Other** (region): Atlantic, Eastern, and Pacific; Other*** (insurer): Insurer 1-4 are the most common insurers in our sample; however, names are not disclosed/specified due to confidentiality. All values are presented as absolute and relative (%) frequencies.
