# Supplementary material for: Teaching hospitals and their influence on survival after valve replacement procedures: A retrospective cohort study using inverse probability of treatment weighting (IPTW)
Source: PLoS One. 2023 Aug 25;18(8):e0290734. doi: 10.1371/journal.pone.0290734 (PMC10456128; doi:10.1371/journal.pone.0290734)
Supplement: S1 Text — (PDF) [file pone.0290734.s007.pdf]

S1 Text. R session.

```
## Analyses were conducted using the R Statistical language (version 4.0.3; R Core
## Team, 2020) on macOS Big Sur 10.16, using the packages DescTools (version
## 0.99.42; Andri et mult. al. S, 2021), gridExtra (version 2.3; Auguie B, 2017),
## Matrix (version 1.3.3; Bates D, Maechler M, 2021), lubridate (version 1.7.10;
## Grolemund G, Wickham H, 2011), Hmisc (version 4.6.0; Harrell Jr F, 2021), rms
## (version 6.2.0; Harrell Jr FE, 2021), arsenal (version 3.6.3; Heinzen E et al.,
## 2021), SurvRegCensCov (version 1.5; Hubeaux S, Rufibach K, 2022), biostat3
## (version 0.1.6; Karlsson A, Clements M, 2021), ggpubr (version 0.4.0;
## Kassambara A, 2020), survminer (version 0.4.9; Kassambara A et al., 2021),
## SparseM (version 1.81; Koenker R, 2021), ResourceSelection (version 0.3.5; Lele
## SR et al., 2019), jtools (version 2.1.4; Long JA, 2020), survey (version 4.1.1;
## Lumley T, 2020), table1 (version 1.4.2; Rich B, 2021), lattice (version
## 0.20.44; Sarkar D, 2008), ipwCoxCSV (version 1.0; Shu D, Wang R, 2019),
## gtsummary (version 1.4.2; Sjoberg D et al., 2021), ggfortify (version 0.4.13;
## Tang Y et al., 2016), survival (version 3.2.11; Therneau T, 2021), ipw (version
## 1.0.11; van der Wal WM, Geskus RB, 2011), MASS (version 7.3.56; Venables WN,
## Ripley BD, 2002), reshape2 (version 1.4.4; Wickham H, 2007), ggplot2 (version
## 3.3.5; Wickham H, 2016), tidyr (version 1.1.4; Wickham H, 2021), dplyr (version
## 1.0.7; Wickham H et al., 2021), knitr (version 1.33; Xie Y, 2021), tableone
## (version 0.13.2; Yoshida K, Bartel A, 2022), Formula (version 1.2.4; Zeileis A,
## Croissant Y, 2010) and sandwich (version 3.0.1; Zeileis A et al., 2020).
##
## References
## ----------
##   - Andri et mult. al. S (2021). _DescTools: Tools for DescriptiveStatistics_.
R
## package version 0.99.42, <URL:https://cran.r-project.org/package=DescTools>.
##   - Auguie B (2017). _gridExtra: Miscellaneous Functions for "Grid"Graphics_. R
## package version 2.3, <URL:https://CRAN.R-project.org/package=gridExtra>.
##   - Bates D, Maechler M (2021). _Matrix: Sparse and Dense Matrix Classesand
## Methods_. R package version 1.3-3,
## <URL:https://CRAN.R-project.org/package=Matrix>.
##   - Grolemund G, Wickham H (2011). "Dates and Times Made Easy withlubridate."
## _Journal of Statistical Software_, *40*(3), 1-25.
## <URL:https://www.jstatsoft.org/v40/i03/>.
##   - Harrell Jr F (2021). _Hmisc: Harrell Miscellaneous_. R package version4.6-0
,
## <URL: https://CRAN.R-project.org/package=Hmisc>.
##   - Harrell Jr FE (2021). _rms: Regression Modeling Strategies_. R packageversi
on
## 6.2-0, <URL: https://CRAN.R-project.org/package=rms>.
##   - Heinzen E, Sinnwell J, Atkinson E, Gunderson T, Dougherty G (2021)._arsenal
:
## An Arsenal of 'R' Functions for Large-Scale StatisticalSummaries_. R package
## version 3.6.3, <URL:https://CRAN.R-project.org/package=arsenal>.
##   - Hubeaux S, Rufibach K (2022). _SurvRegCensCov: Weibull Regression for
## aRight-Censored Endpoint with Interval-Censored Covariate_. R packageversion
## 1.5, <URL: https://CRAN.R-project.org/package=SurvRegCensCov>.
##   - Karlsson A, Clements M (2021). _biostat3: Utility Functions, Datasetsand
## Extended Examples for Survival Analysis_. R package version 0.1.6,<URL:
## https://CRAN.R-project.org/package=biostat3>.
##   - Kassambara A (2020). _ggpubr: 'ggplot2' Based Publication Ready Plots_.R
## package version 0.4.0, <URL:https://CRAN.R-project.org/package=ggpubr>.
##   - Kassambara A, Kosinski M, Biecek P (2021). _survminer: Drawing SurvivalCurv
es
## using 'ggplot2'_. R package version 0.4.9,
## <URL:https://CRAN.R-project.org/package=survminer>.
##   - Koenker R (2021). _SparseM: Sparse Linear Algebra_. R package version1.81,
## <URL: https://CRAN.R-project.org/package=SparseM>.
##   - Lele SR, Keim JL, Solymos P (2019). _ResourceSelection: ResourceSelection
## (Probability) Functions for Use-Availability Data_. R packageversion 0.3-5,
```

```
## <URL:https://CRAN.R-project.org/package=ResourceSelection>.
##   - Long JA (2020). _jtools: Analysis and Presentation of Social ScientificData
_.
## R package version 2.1.0, <URL:https://cran.r-project.org/package=jtools>.
##   - Lumley T (2020). "survey: analysis of complex survey samples." Rpackage
## version 4.0.Lumley T (2004). "Analysis of Complex Survey Samples." _Journal
## ofStatistical Software_, *9*(1), 1-19. R package verson 2.2.Lumley T (2010).
## _Complex Surveys: A Guide to Analysis Using R: A Guideto Analysis Using R_.
## John Wiley and Sons.
##   - R Core Team (2020). _R: A Language and Environment for StatisticalComputing
_.
## R Foundation for Statistical Computing, Vienna, Austria.<URL:
## https://www.R-project.org/>.
##   - Rich B (2021). _table1: Tables of Descriptive Statistics in HTML_. Rpackage
## version 1.4.2, <URL:https://CRAN.R-project.org/package=table1>.
##   - Sarkar D (2008). _Lattice: Multivariate Data Visualization with R_.Springer
,
## New York. ISBN 978-0-387-75968-5, <URL:http://lmdvr.r-forge.r-project.org>.
##   - Shu D, Wang R (2019). _ipwCoxCSV: Inverse Probability Weighted CoxModel wit
h
## Corrected Sandwich Variance_. R package version 1.0,
## <URL:https://CRAN.R-project.org/package=ipwCoxCSV>.
##   - Sjoberg D, Curry M, Hannum M, Larmarange J, Whiting K, Zabor E
## (2021)._gtsummary: Presentation-Ready Data Summary and Analytic ResultTables_.
## R package version 1.4.2, <URL:https://CRAN.R-project.org/package=gtsummary>.
##   - Tang Y, Horikoshi M, Li W (2016). "ggfortify: Unified Interface toVisualize
## Statistical Result of Popular R Packages." _The R Journal_,*8*(2), 474-485.
## doi: 10.32614/RJ-2016-060 (URL:https://doi.org/10.32614/RJ-2016-060),
## <URL:https://doi.org/10.32614/RJ-2016-060>.Horikoshi M, Tang Y (2018).
## _ggfortify: Data Visualization Tools forStatistical Analysis Results_.
## <URL:https://CRAN.R-project.org/package=ggfortify>.
##   - Therneau T (2021). _A Package for Survival Analysis in R_. R packageversion
## 3.2-11, <URL: https://CRAN.R-project.org/package=survival>.Terry M. Therneau,
## Patricia M. Grambsch (2000). _Modeling SurvivalData: Extending the Cox Model_.
## Springer, New York. ISBN 0-387-98784-3.
##   - van der Wal WM, Geskus RB (2011). "ipw: An R Package for InverseProbability
## Weighting." _Journal of Statistical Software_, *43*(13),1-23. <URL:
## http://www.jstatsoft.org/v43/i13/>.
##   - Venables WN, Ripley BD (2002). _Modern Applied Statistics with S_,Fourth
## edition. Springer, New York. ISBN 0-387-95457-0,
## <URL:https://www.stats.ox.ac.uk/pub/MASS4/>.
##   - Wickham H (2007). "Reshaping Data with the reshape Package." _Journalof
## Statistical Software_, *21*(12), 1-20. <URL:http://www.jstatsoft.org/v21/i12/>.
##   - Wickham H (2016). _ggplot2: Elegant Graphics for Data
## Analysis_.Springer-Verlag New York. ISBN 978-3-319-24277-4,
## <URL:https://ggplot2.tidyverse.org>.
##   - Wickham H (2021). _tidyr: Tidy Messy Data_. R package version 1.1.4,<URL:
## https://CRAN.R-project.org/package=tidyr>.
##   - Wickham H, François R, Henry L, Müller K (2021). _dplyr: A Grammar ofData
## Manipulation_. R package version 1.0.7,
## <URL:https://CRAN.R-project.org/package=dplyr>.
##   - Xie Y (2021). _knitr: A General-Purpose Package for Dynamic ReportGeneratio
n
## in R_. R package version 1.33, <URL:https://yihui.org/knitr/>.Xie Y (2015).
## _Dynamic Documents with R and knitr_, 2nd edition.Chapman and Hall/CRC, Boca
## Raton, Florida. ISBN 978-1498716963, <URL:https://yihui.org/knitr/>.Xie Y
## (2014). "knitr: A Comprehensive Tool for Reproducible Research inR." In Stodden
## V, Leisch F, Peng RD (eds.), _Implementing ReproducibleComputational Research_.
## Chapman and Hall/CRC. ISBN 978-1466561595,<URL:
## http://www.crcpress.com/product/isbn/9781466561595>.
##   - Yoshida K, Bartel A (2022). _tableone: Create 'Table 1' to DescribeBaseline
## Characteristics with or without Propensity Score Weights_. Rpackage version
## 0.13.2, <URL:https://CRAN.R-project.org/package=tableone>.
```

```
##   - Zeileis A, Croissant Y (2010). "Extended Model Formulas in R: MultipleParts
## and Multiple Responses." _Journal of Statistical Software_,*34*(1), 1-13. doi:
## 10.18637/jss.v034.i01 (URL:https://doi.org/10.18637/jss.v034.i01).
##   - Zeileis A, Köll S, Graham N (2020). "Various Versatile Variances:
## AnObject-Oriented Implementation of Clustered Covariances in R." _Journalof
## Statistical Software_, *95*(1), 1-36. doi: 10.18637/jss.v095.i01(URL:
## https://doi.org/10.18637/jss.v095.i01).Zeileis A (2004). "Econometric Computing
## with HC and HAC CovarianceMatrix Estimators." _Journal of Statistical
## Software_, *11*(10), 1-17.doi: 10.18637/jss.v011.i10
## (URL:https://doi.org/10.18637/jss.v011.i10).Zeileis A (2006). "Object-Oriented
## Computation of Sandwich Estimators."_Journal of Statistical Software_, *16*(9),
## 1-16. doi:10.18637/jss.v016.i09 (URL: https://doi.org/10.18637/jss.v016.i09).
```
