# Supplementary material for: Teaching hospitals and their influence on survival after valve replacement procedures: A retrospective cohort study using inverse probability of treatment weighting (IPTW)
Source: PLoS One. 2023 Aug 25;18(8):e0290734. doi: 10.1371/journal.pone.0290734 (PMC10456128; doi:10.1371/journal.pone.0290734)
Supplement: Attachment — Submitted filename: Response to Reviewers1.pdf [file pone.0290734.s008.pdf]

Redoy Ranjan, MBBS, MRCSEd, Ch.M., MS (CV&TS), FACS
Academic Editor
PLOS ONE

Subject: Submission of the revised manuscript [PONE-D-23-15501 entitled "*Teaching Hospitals and Their Influence on Survival After Valve Replacement Procedures: A Retrospective Cohort Study using the IPTW*"

Dear Dr. Ranjan,

We thank you and the reviewers for a thorough and careful reading of our work and for the thoughtful comments and constructive suggestions that helped improve the quality of this manuscript.

In the following, we respond to individual remarks in a point-by-point manner. Reviewers' comments are identified by 'RC' and authors by 'AC'. Changes in the manuscript are highlighted in a separate file labeled *'Revised manuscript with track changes'.*

We hope the revised version is now suitable for publication, and we look forward to hearing from you in due course.

Sincerely,

Kevin Maldonado-Cañón
Corresponding author
On behalf of all co-authors

Journal Requirements

1. Please ensure that your manuscript meets PLOS ONE's style requirements, including those for file naming.

AC1: As indicated, we adjusted our manuscript to meet the style requirements of PLOS ONE according to the 'MANUSCRIPT BODY FORMATTING GUIDELINES' and 'TITLE, AUTHOR, AFFILIATIONS FORMATTING GUIDELINES' documents.

---

2. In your Data Availability statement, you have not specified where the minimal data set underlying the results described in your manuscript can be found. PLOS defines a study's minimal data set as the underlying data used to reach the conclusions drawn in the manuscript and any additional data required to replicate the reported study findings in their entirety. … If there are ethical or legal restrictions to sharing your data publicly, please explain these restrictions in detail.

AC2: Thank you for your comment. Unfortunately, due to legal restrictions, we are unavailable to share the data publicly. According to the journal requirements, we have included this information in the Data Availability statement, which now reads:

"Data cannot be shared publicly because of a confidentiality agreement between Universidad Nacional de Colombia and the Ministry of Health of Colombia. Data are available from the integrated information system of the Ministry of Health of Colombia (https://www.sispro.gov.co/Pages/Home.aspx) for researchers who meet the criteria for access to confidential data."

---

3. Please include captions for your Supporting Information files at the end of your manuscript, and update any in-text citations to match accordingly.

AC3: We have included captions at the end of our manuscript and updated in-text citations to match our Supporting Information files. Supporting Information files are also re-uploaded separately (as indicated by PLOS ONE's style requirements).

Reviewer #1

RC1: The authors state that using conventional PS matching was not feasible. How many matched pairs were they able to identify and what was the between group baseline discrepancies?

AC1: Thank you for your comment. Information on between-group baseline discrepancies (reflecting those variables included in the propensity score model for the inverse probability of treatment weighting (IPTW)) is provided in Table 1.

Regarding the "matched pairs," the IPTW provides a weighted sample instead of a whole number of matched pairs. The weighting provided a representation of 1,889.5 and 1,935.6 patients for the teaching and non-teaching hospital groups, respectively. The sample characteristics before and after IPTW are provided in S6 Table. According to this comment, the final paragraph of the 'IPTW' subsection within the 'Results' section has been adjusted and now reads:

"IPTW with restriction provided a representation of 1,889.5 and 1,935.6 patients for the teaching and non-teaching hospital groups, respectively; the largest standardized difference was 7.6% (variable "EPS"), with no values greater than 10%. This diagnosis suggests that the IPTW with restriction allows us to create a weighted artificial balanced sample (sample characteristics before and after IPTW are provided in S6 Table)." (Pg. 11)

RC2: Provide data in tables per group (teaching vs non teaching)

AC2: As suggested, information per group (teaching / non-teaching) is provided in separate columns in Tables 1, S2, and S5. Further, an additional S3 Table is included (*Cumulative incidences of death at 30 days, 90 days, and one year per teaching hospital status*). Also, the 'Exposure' (i.e., teaching / non-teaching) variable is provided in Tables 2 and S4.

RC3: The quality of the figures are low. Could you please provide higher quality ones?

AC3: Thank you for pointing this out. High-quality figures are provided (tiff file format, 300 dpi, Width: 2250 pixels, Height: 900 pixels).

Reviewer #2

RC1: The manuscript needs an English revision.

AC1: Thank you for your comment. We conducted a thorough review and editing of the manuscript to achieve more clear and effective communication of our work.

RC2: In the title the authors wrote "Valve Replacement Procedures" but they included in the analysis also the valve repair. It could be necessary to change the title.

AC2: Thank you for pointing this out. As described in the Materials and Methods section, we included in the analysis patients who "*underwent an isolated or combined valve replacement procedure between January 1, 2016, and December 31, 2019 (index procedure)*". Patients who underwent an isolated **valve repair** procedure were not considered for our study.

Data on **valve repair** provided in the S2 table correspond to those other concomitant cardiovascular surgical procedures performed simultaneously with the index valve replacement or as a reintervention.

Table S2 is adjusted to separately show the frequencies of valve replacement procedures and other concomitant cardiovascular surgical procedures performed simultaneously with the 'index procedure' and the frequencies of valve reinterventions.

RC3: The authors included in the analysis all the intervention techniques (traditional surgery, minimally invasive surgery, transcatheter procedures), the treatment of all heart valve and the single or combined procedures. To better understand the difference between teaching and no- teaching hospitals, it would be better to focus on a single valve procedure or on a single type of intervention technique.

AC3: This is a valid point that we have also given a lot of thought to while working on the paper. Our argument for including this broad range of techniques, valves, and intervention weights (isolated or combined) was that in our region, no studies had encompassed an array of major valve replacement procedures that span the breadth of care on a national scale. We trust that this paper will be an opportunity to provide such data to the public and will be of great benefit, encouraging further research on this topic.

Additionally, incorporating variables such as *Technique* and *Weight of procedure* in the Propensity Score specification model and in the balance assessment after IPTW would reduce potential confounding bias and allow strong conclusions. We have added the following lines addressing this point to the Discussion section:

"Finally, one could argue that including such a diverse set of procedures and techniques would mislead the interpretation of the results. Nonetheless, the main push of our study was to provide previously unreported data enclosing a comprehensive array of major valve replacement procedures transverse to the extent of health care on a national scale; therefore, we included all valve replacements disregarding the treated valve, the weight of the procedure and the technique. … Further prospective, valve- and technique-specific studies should confirm the conclusions drawn from our work." (Pg. 15)

RC4: The primary source of information was the Capitation Sufficiency Database from the 94 Colombian Ministry of Health and the patient's data were assumed from demographic information and the associated ICD-10 codes. This has meant that much information regarding preoperative risk factors and the clinical course of patients is not available for analysis. Although the authors have included this topic in the limitations of the study, it's an important shortcoming that should be filled.

AC4: Thank you for your comment. We have also considered this point while working on the paper. Unfortunately, preoperative risk factors and specific data on patient clinical course are not recorded in the databases that we have available in our country.

Nonetheless, several factors reinforce the validity of both our methods and our results: First, our database has been used in previous studies with similar outcomes that have controlled the effect of possible unobservable confounders through the design by considering attributes such as age, sex, Charlson Comorbidity Index (CCI) score, technique and weight of the procedure, year of surgery, region, and insurer as a proxy for the preoperative and clinical complexity of the case [a-f]. Second, we calculated the CCI using a method that has been shown to be valid once applied to our same database for 1-year mortality [g], thus providing a broad range of preoperative risk factor variables that have been previously used within the context of heart surgery. And third, our descriptive and inferential analysis results were consistent with prior literature, as depicted in the discussion section.

Ref.

a) Buitrago G, Junca E, Eslava-Schmalbach J, Caycedo R, Pinillos P, Leal LC. Clinical Outcomes and Healthcare Costs Associated with Laparoscopic Appendectomy in a Middle-Income Country with Universal Health Coverage. World J Surg. 2019;43(1):67-74. doi:10.1007/s00268-018-4777-5

b) Valero J, Buitrago G, Eslava-Schmalbach J, Rincon CJ. Prognostic Factors Associated with Clinical and Economic Outcomes of Appendectomies in Children: A Multilevel Analysis in a National Retrospective Cohort Study. World J Surg. 2020;44(1):303-312. doi:10.1007/s00268-019-05182-w

c) Buitrago G, Ortiz JR, Camacho F. Clinical Outcomes, Health Care Costs and Prognostic Factors for Total Knee Arthroplasty: A Multilevel Analysis of a National Cohort Study Using Administrative Claims Data. J Knee Surg. 2022;35(4):384-392. doi:10.1055/s-0040-1715097

d) Gamboa Ó, Buitrago G, Patiño AF, et al. Fragmentation of Care and Its Association With Survival and Costs for Patients With Breast Cancer in Colombia. JCO Glob Oncol. 2023;9:e2200393. doi:10.1200/GO.22.00393

e) Oliveros H, Buitrago G. Effect of renal support therapy on 5-year survival in patients discharged from the intensive care unit. J Intensive Care. 2020;8:63. Published 2020 Aug 18. doi:10.1186/s40560-020-00481-0

f) Oliveros H, Buitrago G. Acute Kidney Injury in a Cohort of Critical Illness Patients Exposed to Non-Steroidal Anti-Inflammatory Drugs. Pharmaceuticals (Basel). 2022;15(11):1409. Published 2022 Nov 14. doi:10.3390/ph15111409

g) Oliveros H, Buitrago G. Validation and adaptation of the Charlson Comorbidity Index using administrative data from the Colombian health system: retrospective cohort study. BMJ Open. 2022;12(3):e054058. Published 2022 Mar 23. doi:10.1136/bmjopen-2021-054058

RC5: In the "Discussion" section, the authors focused the discussion on comparing their mortality rates with the results in the literature. I suggest to better argue the topic of the study, namely the difference in the outcomes between the teaching and no- teaching hospitals.

AC5: We appreciate this comment. We agree with the focus reorientation of what has been proposed. To address these points, we added the following lines to the 'Discussion' section:

"These results are consistent with those observed in earlier studies. In 1991, Sethi et al. reported for 957 valve replacements an operative mortality of 7.2% and 9.7% for those with (49.5%) and without resident assistance, respectively, without finding statistically significant differences when adjusted for specific risk factors (9). Telila et al. in 2017, for 33,790 TAVIs in the US -89.3% performed in teaching hospitals- found no differences in adjusted mortality or severe cardiovascular events, yet an increase in acute kidney injury as an adverse event (OR 1.34, 95% CI 1.04-1.72), a longer length-of-stay (7.7 vs. 6.8 days) and higher average hospitalization costs (USD 50,814 vs. 48,787, p-value=0.02) in teaching hospitals (10). Further, in 2017 Zack et al., for 5,005 tricuspid valve procedures (replacement and repair), did not observe any differences in adjusted mortality for the 86% of patients operated on in a teaching hospital (8).

On the other hand, we found that patients operated on at a teaching hospital had a lower adjusted 30-day mortality risk. This finding broadly supports the work of other studies. In 2019 Shah et al. reported that the teaching hospital status positively affected the adjusted in-hospital mortality for AVR and mitral valve procedures (replacement or repair) and refuted the "July effect" (i.e., the hypothesis that the outcomes of surgeries are worse in the first month of training in which there is a new cohort of residents) for 470,005 cardiovascular surgery procedures (7). Pant et al. in 2016 reported lower in-hospital complications rates in teaching hospitals (42 vs. 50%; p-value <0.001) for 7,405 TAVI in the US in 2012 (88% performed in teaching hospitals) (3). Similarly, Gopaldas et al. in 2012 also reported lower in-hospital complication rates in teaching hospitals for combined AVR and coronary artery bypass grafting (CABG) procedures (5)." (Pgs.13-14)

Reviewer #3

RC1: Although the manuscript is well written, it must be clearer. I need help understanding the aims and the scope of this study. … The Aims of the study should be declared and uniformed in the background of the abstract and the Introduction's end.

AC1: Thank you for pointing this out. We edited the whole manuscript to communicate our work in a more clear and effective manner. In particular, the last paragraph of the Introduction has been adjusted and now reads:

"Considering the knowledge gap in Latin America in this regard, and the ethical and practical unfeasibility that would demand randomly assigning a patient to care in a teaching or non-teaching hospital in a clinical trial, we use the inverse probability of treatment weighting (IPTW) to assess the marginal effect of the teaching hospital status on the survival of patients affiliated with the contributory regime in Colombia who underwent a first heart-valve replacement between 2016 and 2019." (Pg. 4)

Similarly, the Background of the abstract reads:

"The effect of the teaching hospital status on cardiovascular surgery has been of common interest in recent decades, yet its magnitude on heart valve replacement is still a matter of debate. Given the ethical and practical unfeasibility of randomly assigning a patient to such an exposure, we use the inverse probability of treatment weighting (IPTW) to assess this marginal effect on the survival of Colombian patients who underwent a first heart-valve replacement between 2016 and 2019." (Pg. 2)

RC2: The title should be rephrased; IPTW should be elucidated.

AC2: Thank you for your comment. As suggested, IPTW is elucidated in the title, which now reads:

Teaching hospitals and their influence on survival after valve replacement procedures:
A retrospective cohort study using inverse probability of treatment weighting (IPTW)

RC3: The conclusions should present what the study really found and not stating that "present a first step..." and phrases like that.

AC3: Thank you for your comment. We adjusted the conclusion section and now it reads:

"After IPTW, 30-day mortality odds are lower for those operated on in a teaching hospital. There was no effect on survival, 90-day or one-year mortality, 90-day readmission, or one-year reintervention. This study encourages using causal-inference approaches applied to administrative claims data to assess questions that would be otherwise unethical and unfeasible. Taken together, we offer an opening for investigating an exposure that has yet to be explored in Latin America with potential value not only for cardiovascular medicine but also for understanding the teaching hospitals as the essential nature of reality of an academic-clinical synergy." (Pg. 15)

RC4: Finally, in the "Background", both abstract and text, it should be clarified why IPTW is essential.

AC4: As suggested, the reason behind the use of IPTW is clarified in the 'Background' section of the abstract and in the Introduction section. We have adjusted the corresponding text addressing this point as requested in your first comment (please see the text there).
